# Influence of Annealing Atmosphere on the Characteristics of Ga_2_O_3_/4H-SiC n-n Heterojunction Diodes

**DOI:** 10.3390/ma13020434

**Published:** 2020-01-16

**Authors:** Young-Jae Lee, Michael A. Schweitz, Jong-Min Oh, Sang-Mo Koo

**Affiliations:** Department of Electronic Materials Engineering, Kwangwoon University, 20 Kwangwoon-ro, Nowon-gu, Seoul 01897, Korea; yjmjcj@naver.com (Y.-J.L.); michael.schweitz@schweitzlee.com (M.A.S.); jmOh@kw.ac.kr (J.-M.O.)

**Keywords:** gallium oxide, silicon carbide, heterojunction diodes, thermal activation energy

## Abstract

Ga_2_O_3_/4H-SiC n-n isotype heterojunction diodes were fabricated by depositing Ga_2_O_3_ thin films by RF magnetron sputtering. The influence of annealing atmosphere on the film quality and electrical properties of Ga_2_O_3_ layers was investigated. X-ray diffraction (XRD) analysis showed a significant increase in the peak intensities of different faces of β-Ga_2_O_3_ {(−201), (−401) and (002)}. X-ray photoelectron spectroscopy (XPS) measurement showed that the atomic ratio of oxygen increases under high-temperature annealing. Moreover, an N_2_-annealed diode exhibited a greater rectifying ratio and a lower thermal activation energy owing to the decrease in oxygen-related traps and vacancies on the Ga_2_O_3_ film and Ga_2_O_3_–metal interface.

## 1. Introduction

Wide bandgap (WBG) semiconductors find applications in high-power transistors and light detectors. Among the more promising WBG materials, gallium oxide (Ga_2_O_3_) is uniquely transparent to visible and ultraviolet light [1,2,3]. It has a bandgap ranging from ~4.6 to ~4.9 eV, resulting in a high electric breakdown field strength of ~8 MV/cm. The Baliga’s figure of merit (BFOM) of Ga_2_O_3_ is 3400, which is roughly four times higher than that of gallium nitride [4,5]. Ga_2_O_3_ has five crystalline modifications (α, β, γ, δ, and ε), among which the monoclinic β-phase is most stable. Metastable Ga_2_O_3_ films can be obtained by thermal annealing and can be subsequently converted into β-Ga_2_O_3_ in a relatively convenient manner. Ga_2_O_3_ is natively n-doped in the range of 10^16^–10^18^ cm^−3^ due to oxygen vacancies and can be further n-doped to free carrier densities by adding Si, Sn, or Ge [6,7,8,9,10,11]. 

Recrystallization through thermal annealing helps reduce oxygen-related charge traps and is generally an effective method for improving the quality of Ga_2_O_3_ [12,13]. Therefore, investigating the annealing process for Ga_2_O_3_ is a promising research direction. Polycrystalline Ga_2_O_3_ films on glass or sapphire substrates have been converted from amorphous phase through high-temperature annealing [14,15,16]. Hexagonal silicon carbide (4H-SiC; bandgap of ~3.26 eV) can be used as a substrate to grow β-Ga_2_O_3_ layers [17]. Hexagonal silicon carbide (a, b = 3.10 Å and c = 10.12 Å) has a low lattice mismatch of ~2 % with Ga_2_O_3_ (a = 12.33 Å, b = 3.04 Å, and c = 5.80 Å). It also and exhibits a higher thermal conductivity (~4.5 W/cm·°C) than other WBG materials such as GaN (~1.3 W/cm·°C) and Ga_2_O_3_ (0.5 W/cm·°C), making it a suitable substrate for high power applications. 

In this work, heterojunction diodes were fabricated by depositing Ga_2_O_3_ on a 4H-SiC substrate and annealing the diodes under different annealing gases. The effects of the applied annealing gas on the material properties of the resulting Ga_2_O_3_ thin films and the electrical performance of the diodes manufactured from this material are investigated.

## 2. Materials and Methods 

**Figure 1:**
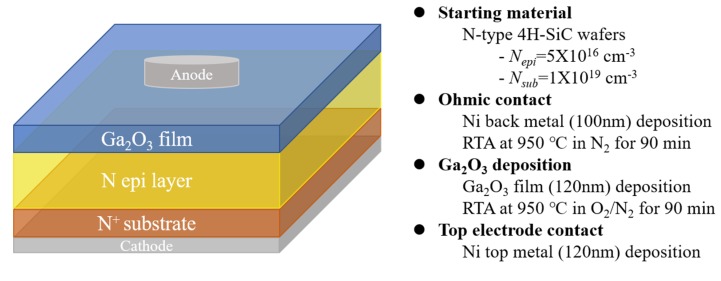
Structure of Ga_2_O_3_/4H-SiC heterojunction diode and fabrication flow.

As a substrate for the gallium oxide film, we used a n-type 4H-SiC wafer (doping concentration: 5 × 10^16^ cm^−3^), with a layer of epitaxially grown 4H-SiC (n-type; 1.0 × 10^19^ cm^−3^), as shown in Figure 1. After cleaning the SiC wafer with SPM solution (H_2_SO_4_:H_2_O_2_ = 4:1), we stripped the native silicon dioxide (SiO_2_) layer using a buffered oxide etch (BOE). A 200-nm-thick nickel film cathode was formed on the bottom side of the SiC wafer by E-beam evaporation. After Ni deposition, the samples were annealed at 950 °C in N_2_ for 10 min by rapid thermal annealing (RTA) for forming ohmic contacts. Gallium oxide thin films were then deposited by radio frequency (RF) sputtering of a Ga_2_O_3_ (99.99% purity) target. Before deposition, the chamber was evacuated to 2.0 × 10^−6^ Torr. The films were grown on the epitaxial 4H-SiC layer under 35 mTorr at a pure argon mass flow rate of 4.6 sccm. The RF power was 140 W, and the films were deposited on room temperature. The thickness of the deposited films ranged from 100 to 250 nm. The SiC wafers, with the deposited Ga_2_O_3_ films, were annealed at 800 °C for 40 min under different atmospheres (pure oxygen and nitrogen gas). An electrode was formed by deposition of 120 nm of nickel on the Ga_2_O_3_ layer.

## 3. Results

### 3.1. Material Properties

**Figure 2:**
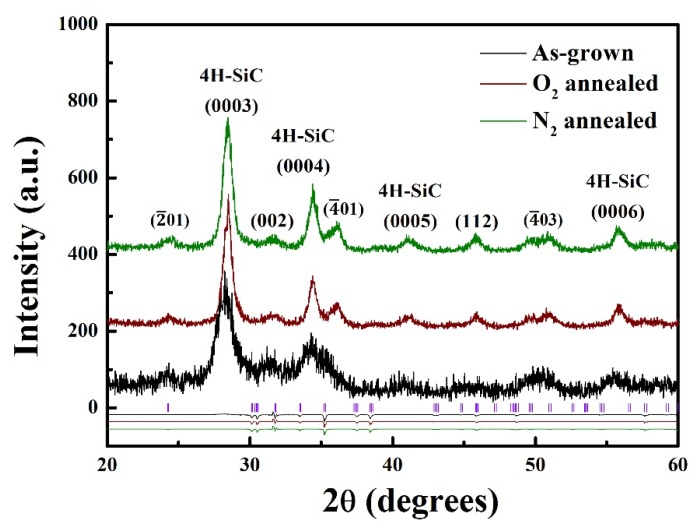
XRD spectra of samples and refinement results of Ga_2_O_3_ annealed under different atmosphere.

To compare the influence of different annealing atmospheres on the crystallinity of Ga_2_O_3_ deposited on the 4H-SiC substrates, X-ray diffraction (XRD) θ–2θ scans were performed on the as-grown, O_2_ and N_2_–annealed samples. As shown in Figure 2, all the sample sets show reflections corresponding to polycrystalline Ga_2_O_3_ with a monoclinic structure from Rietveld refinement by using General Structure Analysis System (GSAS) [18,19]. All the manufactured samples give β-Ga_2_O_3_ diffraction peaks corresponding to (−201), (−401), and (002) faces. The crystal structures remained stable. In fact, the peak intensities were further enhanced after annealing. In particular, the peak intensities corresponding to the (−201) and (−401) faces significantly increased after N_2_ annealing. As explained in the literature, Ga and O atoms migrate under high-temperature annealing and thus help improve the crystallinity of Ga_2_O_3_. Furthermore, dangling bonds related to oxygen defects at grain boundaries can be passivated by N_2_ annealing by incorporating nitrogen atoms at gallium or oxygen lattice sites [20,21]. Consequently, the diffraction peak intensities of the N_2_-annealed samples are higher than those of the other samples, as nitrogen appears to improve the crystal quality of the Ga_2_O_3_ [22,23].

**Figure 3:**
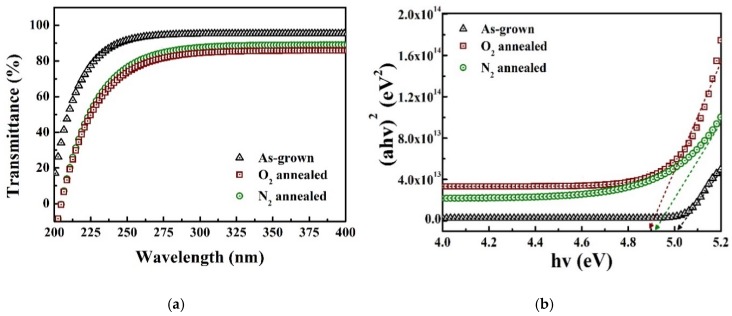
(**a**) Transmittance spectra and (**b**) (αhν)^2^–hν plots of Ga_2_O_3_ film samples annealed under different atmospheres.

Figure 3a shows the optical transmittance spectra of the samples for wavelengths between 200 and 400 nm. All the samples exhibit a high transmittance (over ~80 %) at wavelengths longer than 300 nm. The oxygen concentration in the Ga_2_O_3_ crystals will affect the charge states, which in turn will influence such electrical parameters as bandgap and, consequently, the transmittance [24,25]. The optical bandgap is extracted from the linear part of the graph, shown in Figure 3b, for (αhν)^2^ = 0, where hν is the photon energy, and α is the coefficient of absorption. α = ln(100/T)/d, where T and d is the transmittance and thickness (120 nm) of the Ga_2_O_3_ films, respectively. For the as-grown samples, the bandgap of the Ga_2_O_3_ film is found to be ~5.01 eV. The bandgaps of the samples annealed under O_2_ and N_2_ atmosphere are ~4.91 and ~4.89 eV, respectively. The bandgap of the N_2_-annealed sample is close to the typically reported bandgap value of ~4.9 eV for β-phase Ga_2_O_3_ [26]. 

**Figure 4:**
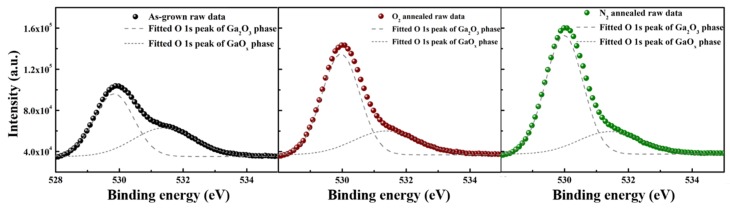
O 1s XPS spectra of the Ga_2_O_3_ films annealed under different atmospheres.

Figure 4 shows the XPS spectra of the O 1s peaks of the three different sample sets. The peaks were calibrated using C 1s at 284.6 eV, in which the O 1s peaks were fitted using two Gaussian peaks, corresponding to Ga_2_O_3_ and GaO_x_ phases, respectively. After annealing in O_2_ and N_2_ atmosphere, the peak intensity of the GaO_x_ phase decreases, whereas that of the Ga_2_O_3_ phase increases. The GaO_x_ peak is reported to have a connection with oxygen vacancies [26]. The magnitude of the peak intensity corresponding to the GaO_x_ phase was reduced, from 37.5% for the as-grown sample to 20.3% and 13.6% for the O_2_ and N_2_-annealed samples, respectively. This is considered to indicate a decrease in the number of defects, such as oxygen vacancies and oxygen sites. The atomic ratios of O to Ga in the samples are 1.40, 1.43 and 1.42 for as-grown, O_2_ and N_2_ annealed Ga_2_O_3_. These different stoichiometric ratios indicate that an increased concentration of O_2_ in the annealing gas can somewhat raise the number of oxygen atoms in the films.

### 3.2. Electrical Properties

**Figure 5:**
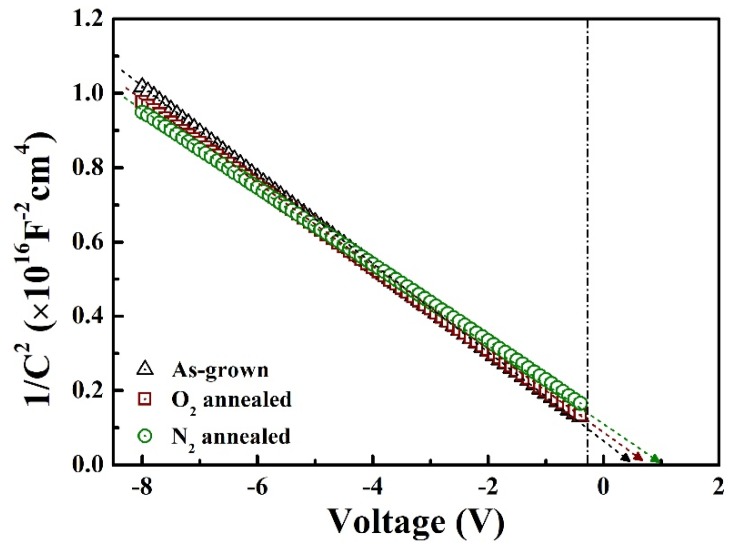
1/C^2^–reverse voltage plots for different diodes measured at room temperature.

Figure 5 shows the graph of 1/C^2^ as a function of the reverse voltage bias applied to the Ga_2_O_3_/4H-SiC diodes. The built-in voltage (V_bi_) and doping concentration can be extracted from the extrapolated graph of 1/C^2^ versus the voltage. V_bi_ is calculated from the V-axis intercept of the fitted graph. The doping concentration is derived from the slope of 1/C^2^–V using Equation (1).

(1) $$\frac{1}{C^{2}}=\frac{2}{qA^{2}}\frac{\varepsilon_{Ga_{2}O_{3}}N_{Ga_{2}O_{3}}+\varepsilon_{SiC}N_{SiC}}{N_{Ga_{2}O_{3}}N_{SiC}\varepsilon_{Ga_{2}O_{3}}\varepsilon_{SiC}}\left(V_{bi}-V\right)$$

The extracted values of V_bi_ and doping concentration of the Ga_2_O_3_ thin films are 0.47, 0.86, and 1.01 V and 9.59 × 10^15^, 1.62 × 10^16^, and 2.01 × 10^16^ cm^−3^ for the as-grown, O_2_-annealed, and N_2_-annealed samples, respectively. The V_bi_ and doping concentration values increased after annealing, because of the decrease in oxygen-related traps. The increase in the built-in voltage can be attributed to the changes in the dopant concentration and the concentration of interface states of Ga_2_O_3_. 

**Figure 6:**
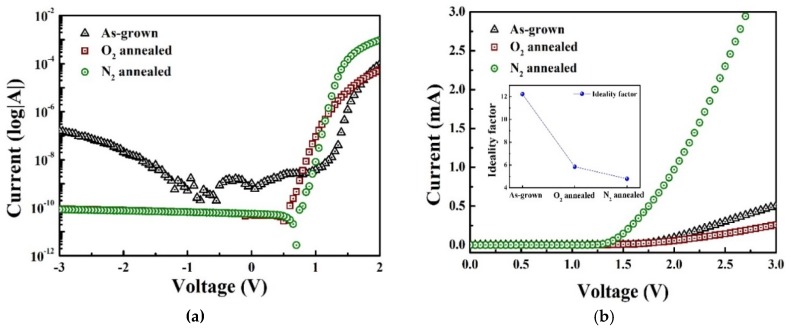
I–V characteristics of diodes at room temperature: (**a**) log-scale and (**b**) linear curves.

Figure 6 shows the typical I–V characteristics of the fabricated Ga_2_O_3_/4H-SiC n-n diodes both in the logarithmic and linear scales. As shown in the figure, the as-grown diode has a high leakage current (~1.60 × 10^−5^ A) and a low rectifying ratio (~3.0 × 10^3^) measured at forward (3 V) and reverse biases (−3 V). The rectifying behavior of the O_2_ and N_2_-annealed diodes is improved. The different samples exhibit a similar leakage current value of approximately 8.1 × 10^−11^ A. The N_2_-annealed diode exhibits a higher on-current when a forward voltage is applied, with a rectifying ratio of ~5.0 × 10^7^, which may be related to the reduced oxygen trap concentrations after annealing. The threshold voltages of the diodes are ~1.55, ~1.47, and ~1.27 V for the as-grown, O_2_ and N_2_-annealed samples, respectively. The ideality factor at room temperature can be extracted from Equation (2).

(2) $$I=I_{O}\left[e^{\frac{V}{\eta K_{B}T}}-1\right]$$

Here, *I* and *V* are the forward current and voltage, respectively, *I_0_* is the saturation current, *k_B_* is Boltzmann’s constant, *T* is the absolute temperature, and *η* is the ideality factor. The ideality factor is significantly reduced after the annealing process; the ideality factor of the N_2_-annealed diode is 2.8, which is half that of the O_2_-annealed diode. The lower ideality factor and the higher built-in voltage of the annealed diodes are attributed to the improved crystallinity and interface properties.

**Figure 7:**
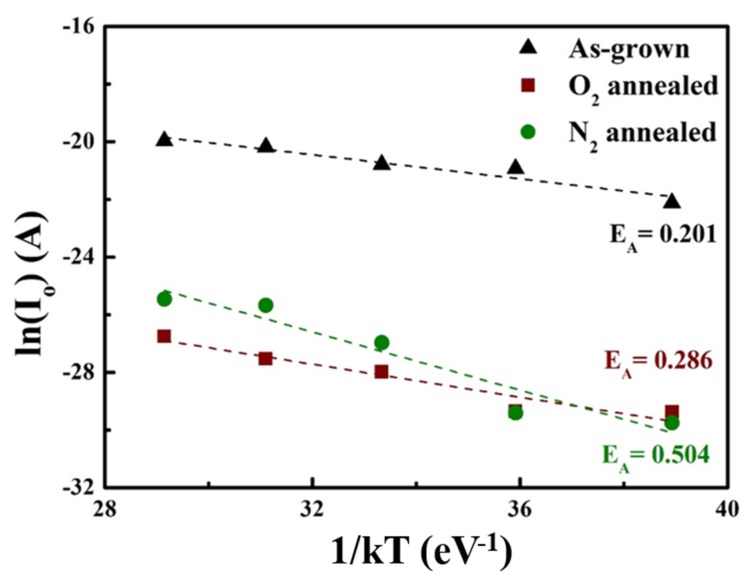
ln(I_O_)–1/kT curve for activation energy values derived from the increasing temperature.

The thermal activation energy (E_A_) is obtained from the ln (I_O_)–1/kT plot shown in Figure 7. The graph was plotted in the temperature range of 298–523 K with a temperature step of 25 K, where I_O_ is the reverse saturation current at −3 V, and *T* and *k* are the absolute temperature and Boltzmann’s constant, respectively. The extracted activation energy from the experimental measurements are related to trap states at the metal–Ga_2_O_3_ interfaces and the barrier heights. Low activation energy values suggest a high concentration of the trap states at the interface, which results in increased trap-assisted tunneling or thermionic emission probabilities across the barrier. As shown in Figure 6, the extracted activation energy of the devices increases after annealing. In particular, the activation energy of the N_2_-annealed sample (0.504 eV) is twice that of the as-grown sample. The improved rectifying ratio of the N_2_-annealed diode is also attributed to the increased activation energy.

## 4. Conclusions

We fabricated polycrystalline β-Ga_2_O_3_/4H-SiC heterojunction diodes annealed under different gas atmospheres (O_2_ and N_2_). The material and electrical properties of the diodes were investigated to understand the effects of the different annealing gases on the device characteristics. X-ray diffraction peaks corresponding to the different faces of β-Ga_2_O_3_ {(−201), (−402), and (002)} were observed to significantly increase, while the bandgap somewhat decreased to ~4.9 eV after annealing. The post-annealing decrease in the GaO_x_ peak intensity indicates a decrease in the number of oxygen vacancies. With regard to the electrical properties, the leakage current decreased nearly 1000 times after annealing. To summarize, the N_2_-annealed sample exhibited higher rectifying ratio and built in voltage, decreased threshold voltage, lower ideality factor, and higher activation energy than the as-grown and O_2_-annealed samples. Therefore, we conclude that the performance of N_2_-annealed diodes at high temperatures is more stable due to higher activation energy compare with built-in voltage due to a lower concentration of trap states.
